# Association between retinal artery lesions and nonalcoholic fatty liver disease

**DOI:** 10.1007/s12072-015-9607-3

**Published:** 2015-02-15

**Authors:** Wen Yang, Hongtao Xu, Xiaohong Yu, Yuzhu Wang

**Affiliations:** 1 Geriatric Digestive System Department, Navy General Hospital, No. 6 Fuchenglu Road, Beijing, 100048 China; 2 Administration Office, Navy General Hospital, Beijing, China

**Keywords:** Nonalcoholic fatty liver disease, Retinal artery lesions, Risk factor

## Abstract

**Objective:**

Retinal artery lesions have been reported to be a risk marker of morbidity and mortality for cardiovascular and cerebrovascular diseases in various study populations. Nonalcoholic fatty liver disease (NAFLD) is also a risk factor for cardiovascular disease. However, the relationship between retinal artery lesions and NAFLD is less certain.

**Methods:**

Data were obtained from 2,454 patients who attended their annual health examination (2,143 males and 311 females, aged 62.34 ± 10.03 years). NAFLD was diagnosed by hepatic ultrasonography. Retinal artery lesions were diagnosed according to the criteria. Total plasma cholesterol, plasma triglyceride and fasting glucose levels were determined by using a multichannel analyzer; the body mass index, systolic blood pressure, diastolic blood pressure, incidence of hypertension and coronary artery disease were measured or analyzed by statistical analysis.

**Results:**

Patients with NAFLD had older age and higher values of BMI, systolic blood pressure, diastolic blood pressure, fasting glucose, total cholesterol and triglycerides and had a higher incidence of hypertension, coronary artery disease and retinal artery lesions (*p* < 0.01). Retinal artery lesions were taken as a dependent variable, and age, gender, BMI, systolic blood pressure, diastolic blood pressure, fasting glucose, total cholesterol and triglyceride levels, and NAFLD were taken as covariates. We found that age ≥65 years (*p* < 0.01, OR 1.968), being male (*p* < 0.01, OR 1.668), BMI ≥ 25 (*p* < 0.01, OR 0.743), SBP ≥ 140 mmHg (*p* < 0.01, OR 1.368) and NAFLD (*p* < 0.01, OR 2.378) were significantly associated with a risk of retinal artery lesions by binary logistic regression analysis.

**Conclusions:**

Patients with NAFLD were older and had higher values for BMI, systolic blood pressure, diastolic blood pressure, fasting glucose, total cholesterol and triglycerides, and higher incidence of hypertension, coronary artery disease and retinal artery lesions. NAFLD is a risk factor for retinal artery lesions.

## Introduction

Nonalcoholic fatty liver disease (NAFLD) has been recognized as the most common liver disease, with an estimated prevalence of 20–30 % [1, 2]. NAFLD includes a spectrum of hepatic dysfunctions ranging from simple steatosis to nonalcoholic steatohepatitis (NASH), cirrhosis and hepatocellular carcinoma [3]. NAFLD is considered to be a hepatic component of metabolic syndrome [4, 5]. It is associated with obesity, dyslipidemia, type 2 diabetes and an increased level of serum fatty acid, and it also predicts the clustering of risk factors for cardiovascular disease [6–8].

Retinal artery lesions are associated with both prevalent and incident metabolic syndrome [9, 10]. It has been demonstrated that retinal artery lesions independently predict incident clinical stroke and lacunar infarcts [11]. Several studies have demonstrated that retinal artery lesions independently predict ischemic heart disease and heart-related death [12].

Other studies have documented a significant association between NAFLD and carotid artery intima media thickness, an early indicator of subclinical atherosclerosis [13, 14]. Retinal artery lesions are part of cerebrovascular atherosclerosis. Nevertheless, the relationship between NAFLD and retinal artery lesions is not clear in large samples, especially in the Chinese population. Therefore, the present study aimed to extensively investigate the association between ultrasonographically diagnosed NAFLD and retinal artery lesions in a large middle-aged and elderly Chinese population.

## Subjects and methods

Study population and data collection: All of the subjects were residents in the community whose health care was provided by our hospital and initially attending their annual health examination in 2012. Inclusion criteria were complete available medical records; normal blood pressure [diastolic blood pressure (DBP) of <90 mmHg and systolic blood pressure (SBP) of <140 mmHg] or well-controlled hypertension (DBP of <90 mmHg and SBP of <150 mmHg); and no smoking or history of alcohol consumption. The hypertensive patients were taking antihypertensive medication, and all the observed subjects were taking acetylsalicylic acid (100 mg/day). Exclusion criteria were the presence of hematological system diseases; past history of cancer; major gastrointestinal surgery, including partial or total gastrectomy or colorectomy; pulmonary disease; nephrotic syndrome or serum creatinine levels higher than 115umol/l; total parenteral nutrition; viral hepatitis, autoimmune hepatitis and liver degeneration, etc.; and taking statin drugs or other drugs that damage the liver. Diabetes mellitus was diagnosed according to the 2009 American Diabetes Association diagnostic criteria [15]. Finally, 2,454 eligible subjects were enrolled (2,143 males and 311 females, aged 62.34 ± 10.03 years). Verbal informed consent was obtained from each subject and was recorded by the physician who explained the study procedures.

### Clinical examination

All patients were required to refrain from exercise for 1 day prior to the examination. Blood pressure was measured in the right arm using a mercury sphygmomanometer after 20 min of rest with the patient in a sitting position. Standing height, body weight and waist circumference were recorded for all subjects. Waist circumference was measured with the measuring tape positioned midway between the lowest rib and the superior border of the iliac crest as the patient exhaled normally. Body mass index (BMI) was calculated as weight divided by height squared.

#### Biochemical analyses

The blood samples were drawn from the participating patients after an overnight fast of more than 12 h. The serum levels of plasma creatinine concentration, blood urea nitrogen, total plasma cholesterol, plasma triglyceride and fasting glucose were measured using a multichannel analyzer (Roche Hitachi 737; Boehringer Mannheim Diagnostics, USA).

### Diagnosis of NAFLD, hypertension and coronary artery disease

The diagnosis of NAFLD was based on the criteria suggested by the Chinese Liver Disease Association [16]. Ultrasonic examination was carried out by a trained ultrasonographist who was unaware of the results of the physical examination and biochemical analyses. Diffuse fatty liver can be defined by abdominal ultrasonography with the presence of at least two of three findings: “bright liver,” liver echo greater than that of the kidney, vascular blurring and the gradual attenuation of far field ultrasound echo [16]. The examination was performed using a Toshiba Nemio 20 sonography machine with a 3.5-MHz probe (Toshiba, Tokyo, Japan).

Hypertension was defined as a SBP ≧140 mmHg or DBP ≧90 mmHg according to the Seventh Report of the Joint National Committee [17], or when the patients had a history of hypertension or were receiving antihypertensive treatment.

The diagnostic criteria for coronary heart disease included patients with previous onset of myocardial infarction, with angina symptoms or ECG changes at the same time of more than 75 % narrowing of the coronary artery confirmed by coronary angiography. The symptoms of angina pectoris include chest pain, dyspnea, diaphoresis and palpitation, and the changes in electrocardiograms include significant ST-T wave change or Q wave presentation.

### Assessment of retinal artery lesions

The eye fundus examination was carried out for all the patients before ultrasonic examination by direct ophthalmoscopy performed after pupil dilation. The ophthalmologists who conducted the ophthalmoscopy did not know the patients' ultrasonic examination results. For all patients, the upper temporal quadrants and the first three arterial branches were analyzed. The extent and severity of atherosclerotic vascular lesions in the retinal arteries were classified according to Scheie. Stage 1 is defined as a broadening of the light reflex from the artery with minimal or no arteriovenous compression. Stage 2 is defined as changes similar to those in stage 1, but more prominent. In stage 3, the arteries have a “copper wire” appearance, the arteriovenous compression is much greater, and serious atherosclerotic changes of the retinal arteries are present. Stage 4 is the most severe form of atherosclerosis of the retinal arteries.

### Statistical analysis

Data were expressed as mean ± SD or counts. Statistical analysis was performed using SPSS version 16.0 (SPSS Inc., Chicago, IL), and the level of statistical significance was defined as *p* < 0.05. The independent-samples *t* test was used for the comparisons of continuous data, while the chi-square test was used for the comparisons of categorical variables. Binary logistic regression analysis was used to determine the factors that were associated with retinal artery lesions.

## Results

### Baseline characteristics

Among the 2,454 enrolled patients (2,143 males and 311 females, aged 62.34 ± 10.03 years), 872 (785 males and 97 females) met the diagnostic criteria for NAFLD; the prevalence of NAFLD was 35.53 % (males 36.63 % and females 31.19 %). The characteristics of the patients, classified by the presence or absence of NAFLD, are presented in Table 1. Patients with NAFLD were older and had higher levels of BMI, SBP, DBP, fasting glucose, total cholesterol and triglyceride levels and had a higher incidence of hypertension, coronary artery disease and retinal artery lesions (*p* < 0.01).

**Table 1:** Characteristics of study subjects according to the presence of NAFLD

Variables	NAFLD absent (*n* = 1,582)	NAFLD present (*n* = 872)	*p* value
Age, years	61.94 ± 9.954	63.06 ± 10.127	0.008
Male, *n*	1,358 (85.84 %)	785 (90.02 %)	0.003
BMI, kg/m^2^	24.31 ± 2.90	26.19 ± 3.14	0.000
SBP, mmHg	130.08 ± 15.38	135.17 ± 14.68	0.000
DBP, mmHg	81.06 ± 8.70	83.82 ± 8.28	0.000
Fasting glucose, mmol/l	5.34 ± 1.19	5.56 ± 1.18	0.000
Total cholesterol, mmol/l	4.76 ± 1.12	4.89 ± 1.15	0.008
Triglycerides, mmol/l	1.35 ± 1.13	1.69 ± 1.37	0.000
Hypertension, *n*	406 (25.66 %)	304 (34.86 %)	0.000
Coronary artery disease, *n*	267 (16.88 %)	189 (21.67 %)	0.004
Diabetes mellitus, *n*	167 (10.56 %)	114 (13.07 %)	0.061
Retinal artery lesions, *n*	463 (29.27 %)	427 (48.97 %)	0.000
Stage 1	344 (21.74 %)	163 (18.69 %)	0.074
Stage 2	119 (7.52 %)	264 (30.27 %)	0.000

Data are presented as mean ± SD unless otherwise indicated

*BMI* body mass index, *SBP* systolic blood pressure, *DBP* diastolic blood pressure

### NAFLD and risk factors for retinal artery lesions

Binary logistic regression analysis was used to evaluate the risk factors for retinal artery lesions. Retinal artery lesions were taken as the dependent variable and age, gender, BMI, SBP, DBP, fasting glucose, total cholesterol, triglyceride, NAFLD and coronary artery disease were taken as covariates. We found that age ≥65 years (*p* < 0.01, OR 1.968), being male (*p* < 0.01, OR 1.668), BMI ≥ 25 (*p* < 0.01, OR 0.743), SBP ≥ 140 mmHg (*p* < 0.01, OR 1.368) and NAFLD (*p* < 0.01, OR 2.378) were significantly associated with the risk of retinal artery lesions (Table 2).

**Table 2:** The results of binary logistic regression analysis on retinal artery lesions

	Variable	SE	Wald	*p* value	OR	95.0 % CI	
						Lower	Upper
Age, years	<65 = 0, ≥65 = 1	0.092	54.434	0.000	1.968	1.644	2.356
Male, *n*	Female = 0, male = 1	0.149	11.875	0.001	1.668	1.247	2.232
BMI, kg/m^2^	<25 = 0, ≥25 = 1	0.094	10.053	0.002	0.743	0.618	0.893
SBP, mmHg	<140 = 0, ≥140 = 1	0.113	7.753	0.005	1.368	1.097	1.707
DBP, mmHg	<90 = 0, ≥90 = 1	0.120	0.053	0.817	0.973	0.769	1.230
Fasting glucose, mmol/l	<6.1 = 0, ≥6.1 = 1	0.118	2.842	0.092	0.819	0.649	1.033
Total cholesterol, mmol/l	<25 = 0, ≥25 = 1	0.110	1.857	0.173	1.161	0.937	1.440
Triglycerides, mmol/l	<5.69 = 0, ≥5.69 = 1	0.101	0.522	0.470	0.929	0.762	1.134
NAFLD, *n*	Absent = 0, present = 1	0.096	82.027	0.000	2.378	1.972	2.868
Coronary artery disease	Absent = 0, present = 1	0.102	0.614	0.433	0.923	0.757	1.127

*CI* confidence interval; dependent variable: retinal artery lesions

## Discussion

The retinal artery is part of the cerebral circulation system and offers an opportunity to explore atherosclerosis in the small peripheral arteries noninvasively. An assessment of the retinal artery may offer the opportunity to determine the extent of atherosclerosis in human body.

NAFLD is a hepatic manifestation of the metabolic syndrome and is closely related to other clinical features of the metabolic syndrome; thus, cardiovascular disease is increased in NAFLD, and NAFLD represents the main cause of death in these patients. Patients with NAFLD have significantly higher rates of prevalent coronary, cerebrovascular and peripheral vascular disease than their counterparts without NAFLD [18–21].

This research is a study on the community residents whose health care was provided by our hospital. In our study, the retinal artery lesions were significantly increased in NAFLD patients; retinal artery lesions were associated with age, being male, BMI, DBP, total cholesterol, triglycerides and NAFLD, showing that NAFLD is associated with cerebral atherosclerosis. Other research [22, 23] has reported that retinal vascular changes are related to NAFLD, which is similar to our results.

The mechanisms underlying the association between retinal artery lesions and NAFLD are currently unclear. The pathogenetic mechanism might include endothelial dysfunction, oxidative stress, inflammation, inflammatory cytokines, dyslipidemia and glucose metabolism disorder [24].

Several studies have demonstrated that metabolic syndrome has important implications for the clinical results of NAFLD patients [25–27] and that advanced forms of NAFLD stimulate increasing insulin resistance and dyslipidemia. In this way, the progression of atherosclerosis is accelerated. In our study, BMI, total cholesterol and triglyceride levels, which are the components of metabolic syndrome, were significantly increased in NAFLD patients.

Oxidative stress and inflammation are associated with retinal artery lesions [26]. Increased oxidative stress and subclinical inflammation, which are considered to be causal factors in the progression from simple steatosis to more advanced forms of NAFLD, may represent a possible atherogenic mechanism linking NAFLD and retinal artery lesions [27, 28].

Retinal artery lesions have been associated with blood pressure [29]. In our research, this was only related to SBP, but not to DBP, which may be because the hypertensive patients who attended our annual health examination tended to be elderly. Our results indicate that coronary heart disease was not associated with retinal artery lesions, which may be because the diagnoses of coronary heart disease were mainly based on the medical history, symptoms and electrocardiograms, and only the subjects suspected of having coronary artery disease were checked by coronary angiography examination, not all of them.

Another possible mechanism linking NAFLD and retinal artery lesions could be decreased plasma levels of adiponectin, an adipose-secreted cytokine with antiatherogenic properties. It has been shown that hypoadiponectinemia closely correlates to NAFLD in obese individuals, unrelated to insulin resistance and other metabolic syndrome components [30, 31]. However, adiponectin plays an active role in the pathophysiology of atherosclerosis [32].

A few limitations warrant consideration. First, the diagnosis of NAFLD was based on ultrasound imaging. The patients did not undergo liver biopsy and histological examination, which is the gold standard technique for identifying steatosis. The sensitivity of ultrasonography in detecting steatosis varies between 60 and 94 % and is dependent on the degree of steatosis. Second, our research was a single-center study; thus, our relatively small sample size may have posed a limitation to the study. Therefore, our findings need to be confirmed in multicenter and prospectively designed studies. Finally, retinal artery lesions were not observed dynamically; thus, it remains unclear whether retinal artery occlusion increases incrementally whether the patient’s condition progressively deteriorates or not.

In conclusion, NAFLD is extremely common in the middle-aged and elderly Chinese population. Patients with NAFLD had older age and higher levels of BMI, SBP, DBP, fasting glucose, and total cholesterol and triglycerides, and they had higher incidences of hypertension, coronary artery disease and retinal artery lesions. NAFLD is a risk factor for retinal artery lesions. NAFLD may be involved in the formation of cerebrovascular atherosclerosis, and the prevention of NAFLD has an effect on the prevention of cerebral vascular disease. Patients with NAFLD should always be assessed for retinal artery lesions to ensure early diagnosis and entry into proper and thorough medical care.
